# The Big Picture

**Published:** 2004-11

**Authors:** Laura Alderson

## Mapping SARS in Hong Kong

Epidemiologists have long used maps to track the spread of disease, and in the past decade, geographic information system (GIS) technology has added powerful new tools that help reveal far more than simply the “where” and “when” of epidemics. Now P.C. Lai of the University of Hong Kong and colleagues show how GIS technology can be used during an acute infectious disease outbreak to reveal crucial real-time, quantitative information, such as the direction of superspreading events (in which one person infects more than the typical three or fewer others) and distinct disease hot spots **[EHP** **112:1550–1556]**. Reaching beyond typical descriptive mapping, this study demonstrates the rich depth of GIS capabilities in analyzing patterns of disease spread from various perspectives.

The global outbreak of severe acute respiratory syndrome (SARS) in late 2002 and into 2003 ultimately accounted for more than 8,000 cases in 29 countries, according to the World Health Organization. About 20% of the cases were in Hong Kong. Lai and colleagues applied geostatistical methods to analyze the spread of SARS in Hong Kong during this time period.

The investigators analyzed an integrated database that contained clinical and personal details on the 1,755 Hong Kong patients confirmed to have had SARS. They plotted patient residence addresses using a GIS to research such aspects as the superspreading event responsible for more than 300 cases in the Amoy Gardens housing development and microclusters of SARS cases (where the density of infection varied widely between districts).

The geostatistical analysis was conducted at three levels: elementary (visual inspection of geographical phenomena), cluster analysis to identify hot spots, and contextual analysis to explain relationships between geographical phenomena. Among the methods the researchers applied were nearest neighbor analysis, which discerns nonrandom distribution of cases and is often used by scientists studying species distribution. For another analysis, they used the kernel mathematical method to create a series of statistical “surfaces” to reveal daily changes in disease hot spots.

Elementary analysis revealed the spread of the disease: a clear clustering of cases in certain districts of the Kowloon peninsula, where Amoy Gardens is located, and in Hong Kong’s New Territories region. Next, cluster analysis produced a series of 12 kernel maps based on date of symptom onset. These maps showed the density of SARS patients (adjusted for underlying population density) on typical days representing different stages of the 16-week outbreak; this demonstrated the development and dissipation of disease hot spots over the course of events. Another sophisticated analysis produced a map that summarized SARS hot spots by infection rate per 1,000 population, indicating that the urban population was at the highest risk.

With contextual analysis, the researchers developed origin-and-destination plots for three superspreading event clusters: Prince of Wales Hospital, Amoy Gardens, and Lower Ngau Tau Kok Housing Estate. The Prince of Wales Hospital cluster showed a northwest–southwest trend of disease spread that extended over most of Hong Kong (visitors to SARS patients at Prince of Wales Hospital spread the disease as they returned home, the authors observed). The Amoy Gardens cluster was comparatively more localized, while the Lower Ngau Tau Kok cluster was the most contained of the three.

The authors cautioned of limitations in applying GIS technology to infectious disease epidemiology and outbreak investigation, among them the occasional lack and unavailability of the necessary data. Still, the authors wrote, “integration of GIS technology into routine field epidemiologic surveillance can offer a scientifically rigorous and quantitative method for identification of unusual disease patterns in real time.” When linked with point-of-care databases and other sources of environmental data (including meteorological, transportation, and topographical information), such geospatial intelligence has the potential to rapidly recognize, locate, and monitor disease outbreaks.

## Figures and Tables

**Figure:**
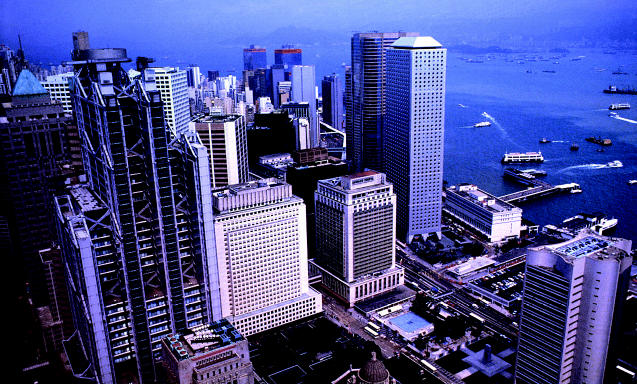
**Bird’s-eye view of SARS.** Using GIS technology, researchers have mapped how SARS spread in Hong Kong to help predict patterns of future infectious disease epidemics.

